# Development of Conjugated Linoleic Acid Nanostructured Lipid Carriers and Their Synergistic Efficacy with Curcumin

**DOI:** 10.3390/foods14173104

**Published:** 2025-09-05

**Authors:** Huan Liu, Xingyu Huang, Yuxiu Liu, Guangming Zheng, Wei Yang, Bo Li

**Affiliations:** 1 School of Food Science, Henan Institute of Science and Technology, Xinxiang 453003, China; lh18211673717@163.com (H.L.); h18567341821@163.com (X.H.); liuyuxiu1028@163.com (Y.L.); z17362900436@163.com (G.Z.); 2 School of Food Science and Engineering, Henan University of Technology, Lianhua Road 100, Zhengzhou 450001, China; 3 State Key Laboratory of Food Science and Resources, Jiangnan University, Wuxi 214122, China

**Keywords:** curcumin, conjugated linoleic acid, encapsulation, nanostructured lipid carrier, antioxidant activity, synergistic efficacy

## Abstract

Curcumin has good anti-cancer and antioxidant properties. However, the poor water solubility and low bioavailability limit its application in food products. This study constructed a nanostructured lipid carrier (Cur-CLA-NLC) encapsulating curcumin using conjugated linoleic acid (CLA) as the liquid lipid and stearic acid as the solid lipid. Cur-CLA-NLC exhibits significantly enhanced bioaccessibility, antioxidant activity, and cytocompatibility. CLA, as a liquid lipid in Cur-CLA-NLC, has a dual role as a structural stabilizer and bioactive agent, and synergistically enhances antioxidant activity with curcumin. In vitro simulated digestion studies showed that the bioaccessibility of curcumin in Cur-CLA-NLC (85.7%) was much higher than that in the pure curcumin (11.7%) and curcumin lipid mixtures (9.3%). In addition, the Cur-CLA-NLC system showed anti-lipid peroxidation ability and good biocompatibility. Therefore, CLA-NLC can serve as a potential delivery system for enhancing health benefits via functional foods.

## 1. Introduction

Curcumin (Cur) is a plant polyphenol extracted from turmeric. As a food additive with health benefits, it also has anti-inflammatory, antioxidant, anti-tumor, and other pharmacological benefits. However, the bioavailability of curcumin is greatly reduced due to the poor water solubility, short cycle half-life, and low absorption rate, which seriously restricts its application in food and drugs [1]. The most commonly used strategy to overcome these solubility problems is encapsulation of curcumin in liposomes, emulsions [2,3], and nanostructured lipid carrier [4], which improves oral bioavailability, pharmacodynamic performance, and reduces toxicity [5].

Nanostructured lipid carrier (NLC) refers to the replacement of part of the solid lipid in solid lipid nanoparticle with liquid lipid (oil), providing sufficient space to accommodate drug molecules and increasing their storage stability. As a drug carrier, NLC has the advantages of strong physical stability and good bioavailability compared with traditional lipid nanocarriers [6,7]. NLC presents increasing potential as a drug carrier to protect unstable drugs from degradation, improve their bioavailability, and control their sustained release [8].

Researchers have made many efforts to try various edible or low-toxic materials to make NLC, such as polymer, wax [9], cholesterol ester, phospholipid for solid lipids [10]; while triglyceride [11] or oleic acid [12] for liquid lipids. Despite extensive research on NLC as drug delivery systems, lipid combinations that simultaneously offer good biocompatibility and added health benefits remain underexplored, particularly for functional food applications.

Conjugated linoleic acid (CLA) is an essential fatty acid that cannot be synthesized by the human body. It has anti-arteriosclerosis, anti-cancer, anti-diabetes, anti-inflammation, anti-oxidation, and other functions [13]. CLA can be used as the main fatty acid to construct various fatty acid vesicles for encapsulation [14]. The CLA, as a liquid lipid, not only addresses formulation compatibility issues but also provides additional health benefits, opening new possibilities for functional food development. Moreover, the potential synergistic effect between CLA and curcumin may further enhance the system’s value.

Herein, a nanostructured lipid carrier (CLA-NLC) with CLA as liquid lipid and stearic acid (SA) as solid lipid was constructed. The encapsulation of the hydrophobic drug curcumin by CLA-NLC and the bioaccessibility, antioxidant activity, and cytocompatibility of Cur-CLA-NLC were investigated. Additionally, synergistic antioxidant activity in the Cur-CLA-NLC system will be verified. This could provide a reference for the nanostructured lipid carrier CLA-NLC to be used as the encapsulation carrier of bioactive substances.

## 2. Materials and Methods

### 2.1. Materials

Curcumin (Cur, 95% of HPLC purity) was from Shanghai Yuanye Bio-Technology Co., Ltd., Shanghai, China. Conjugated linoleic acid (CLA, 95%) was purchased from Da Lian Yinuo Biology Co., Ltd., Liaoning, China. 1,1-Diphenyl-2-picryl hydrazyl radical (DPPH, 98%), 2,2′-azino-bis (3-ethylbenzothiazoline-6-sulfonic acid) (ABTS, 98%), and Thiazolyl Blue Tetrazolium Bromide (MTT, 99%) were from Sigma-Aldrich Co., Ltd., Shanghai, China. Ascorbic acid was from Shanghai Macklin Biochemical Co., Ltd. (99.5 % of purity), Shanghai, China. Trypsin, lipase, pepsin, and bile salt were of BR grade, purchased from Sinopharm Chemical Reagent Co., Ltd., Shanghai, China. Methanol, ethanol, stearic acid (SA), sodium hydroxide (NaOH), Tween 80, and other reagents (analytical grade) were also purchased from Sinopharm Chemical Reagent Co., Ltd., Shanghai, China. The other reagents were purchased from Sinopharm Chemical Reagent Co., Ltd., Shanghai, China. Human normal liver cells L02 and human breast cancer cells MDA-MB-231 were obtained from the cell bank of the Chinese Academy of Sciences, Shanghai, China.

### 2.2. Preparation of the CLA-NLC and Cur-CLA-NLC

The preparation of curcumin-loaded nanostructured lipid carriers is based on the method reported in the literature, with slight modifications [15]. Typically, stearic acid and CLA with different mass ratios were heated to a molten state at 70 °C to form a uniform oil phase. Then, curcumin was added and stirred to dissolve. Tween 80 dissolved in deionized water (0.5–5 wt.%) was heated in a water bath to 70 °C as the water phase. With equal volume of water, the water phase was rapidly added to the oil phase and stirred for 2 min, and then homogenized (T18 digital ULTRA-TURRAX^®^ homogenizer, IKA, Stuttgart, Germany) for 15 min to prepare the emulsion (15,000 rpm, 3 × 5 min). The emulsion was ultrasonically treated for 15 min and cooled to room temperature to obtain the Cur-CLA-NLC suspension. The nanostructured lipid carrier CLA-NLC without curcumin and the physical mixture of curcumin and lipids were used as controls.

### 2.3. Determination of Particle Size and PDI

The particle size and polydispersity index (PDI) of CLA-NLC were determined by ZetaPALS (Brookhaven Instruments Ltd., Holtsville, NY, USA) based on literature methods [16]. CLA-NLC samples were diluted 50-fold with ultrapure water to prevent multiple scattering effects. Disposable polystyrene cuvettes were used as sample cells, equilibrated to 25 ± 0.1 °C. The instrument operates on dynamic light scattering principles, employing a 633 nm laser to detect light scattering intensity from Brownian motion of particles. Diffusion coefficients were calculated using the phase analysis light scattering technique (PALS) and converted to equivalent hydrodynamic diameter via the Stokes–Einstein equation. Each sample was measured three times, and the average value is taken as the average particle size and PDI value, with the measurement angle set at 90°.

### 2.4. Microstructure, Spectral, and Thermal Stability Analysis

#### 2.4.1. Fourier Transform Infrared Spectroscopy (FT-IR) Measurements

The functional groups of molecules in different samples were analyzed by Fourier transform infrared spectroscopy (Nicolet iS50, Thermo Fisher Scientific, Massachusetts, USA) according to the literature [17]. The CLA-NLC and Cur-CLA-NLC samples were freeze-dried at −50 °C for 24 h, and then the freeze-dried samples and curcumin were ground into powder and unrolled on an infrared spectrometer for analysis, with a scanning range of 4000–550 cm^−1^ and 16 scans.

#### 2.4.2. X-Ray Diffraction (XRD) Measurements

The XRD patterns of different samples were analyzed by X-ray diffraction (D8, Bruker AXS, Karlsruhe, Germany) [17]. The samples were prepared by grinding to a fine powder using an agate mortar. The data collection was performed in continuous scan mode with a scanning speed of 4 °C/min in the 2θ range of 5–60°. Triplicate measurements were performed on randomly selected samples, with the relative standard deviation of peak intensities maintained below 5%.

#### 2.4.3. Differential Scanning Calorimeter (DSC) Measurements

The thermal stability of freeze-dried samples was analyzed by a differential scanning calorimeter (204 F1, NETZSCH, Selb, Bavaria, Germany) [14]. For each sample (blank CLA-NLC, Cur-CLA-NLC, SA, curcumin, and Cur-SA-CLA mixture), precisely weighed 5–10 mg aliquots were loaded into high-pressure aluminum crucibles using a microbalance (±0.01 mg precision). Then, under continuous nitrogen purging (70 mL/min), heat dynamically from 25 °C to 220 °C at a constant rate of 10 °C/min. Three complete heating–cooling cycles were performed for each sample to assess thermal reversibility.

#### 2.4.4. Transmission Electron Microscope (TEM) Measurements

The morphology and particle size of CLA-NLC and Cur-CLA-NLC samples were characterized by Transmission Electron Microscope (JEM-2100, Electronics Co., Ltd., Tokyo, Japan) [18]. The TEM samples preparation involved diluting CLA-NLC and Cur-CLA-NLC suspensions 50-fold with phosphate-buffered saline (pH 7.4, 10 mM) to achieve optimal particle dispersion. Using precision tweezers, grids were immersed in the diluted suspension for exactly 2 s, with excess liquid carefully blotted using filter paper. Samples were immediately plunge-frozen in liquid nitrogen for 10 s and transferred to freeze-dry for 24 h. Finally, the samples were observed under a Transmission Electron Microscope at 120 kV.

### 2.5. The Bioaccessibility of Curcumin

The bioaccessibility of curcumin is defined as the proportion of the dissolved fraction in micelles after digestion. The continuous digestion of the stomach–small intestine was simulated sequentially according to literature protocols [19]. Digestion in simulated gastric fluid: A 5 mL sample suspension (curcumin, Cur-SA-CLA, or Cur-CLA-NLC) was combined with 5 mL of electrolyte solution (containing KH_2_PO_4_ and MgCl_2_.6H_2_O). The system pH was adjusted to 4.0 using 1 mol/L HCl, followed by the addition of 5 mL pepsin solution (5.2 mg/mL). After 30 min of dark incubation at 37 °C with shaking, the pH was further reduced to 2.0 with HCl for an additional 30 min digestion period. Following gastric digestion, the sample pH was adjusted to 5.0 using 1 mol/L NaOH, then mixed with 3 mL simulated intestinal fluid containing trypsin (4 mg/mL), lipase (2 mg/mL), and bile salts (2.5 mg/mL) in PBS (phosphate-buffered saline, pH 7.4). The mixture pH was further adjusted to 7.0 and incubated at 37 °C for 2 h with dark shaking. The digestive products were centrifuged (10,000 rpm, 4 °C, 30 min), and the supernatant was collected for spectrophotometric quantification of curcumin content. The transformation, bioaccessibility index, and bioaccessibility of curcumin were calculated according to the following formulas:

(1) $$\mathrm{Transformation}\%\;=\;A_{digesta}/A_{initial}\;\times \;100\%$$

(2) $$\mathrm{Bioaccessibility}\;\mathrm{index}\%=A_{micelle}/A_{digesta}\;\times \;100\%$$

(3) $$\mathrm{Bioaccessibility}\%=A_{micelle}/A_{initial}\;\times \;100\%$$

where *A_digesta_* and *A_micelle_* are the total amount of curcumin in the digestive fluid and micelle, respectively; *A_Initial_* is the initial amount of curcumin in the digestive system.

### 2.6. Determination of Solubility of Curcumin in Lipid Phase

Curcumin exhibits lipid solubility, and studying its solubility in lipid phases is not only a critical parameter for optimizing its delivery systems but also provides a theoretical foundation for expanding its applications in antioxidant and bioaccessibility fields. Determination of standard curve of curcumin ethanol solution: Preparation curcumin ethanol solutions with concentrations of 1 μg/mL, 2.5 μg/mL, 5 μg/mL, 7.5 μg/mL and 10 μg/mL, and the maximum absorption wavelength of the solution was obtained by UV-Vis (Ultraviolet–visible spectroscopy, TU-1901, Beijing Purkinje General Instrument Co., Ltd., Beijing, China), and then the absorbance at the wavelength was determined to obtain the standard curve of absorbance change with concentration.

Curcumin solubility was determined by UV-Vis spectrophotometry using a literature method [20]. Briefly, 10 mg of curcumin was dissolved in 10 g lipid phase (SA-CLA), stirred at 1500 rpm for 45 min in a 65–70 °C water bath, and ultrasonic for 10 min. Then, the lipid phase dissolved curcumin was placed in a water bath at 65–70 °C for 24 h. Finally, 0.5 mL of the upper lipid phase was added to 50 mL of ethanol solution, and the absorbance at the maximum absorption wavelength was measured by an ultraviolet spectrophotometer. The solubility of curcumin in the lipid phase was quantified via a curcumin/ethanol standard curve.

### 2.7. Determination of Antioxidant Activities

#### 2.7.1. DPPH Radical Scavenging Activity

The antioxidant activity of curcumin, CLA-NLC, and Cur-CLA-NLC was evaluated using a modified DPPH assay [21]. Briefly, 1.5 mL test sample was mixed with 4 mL DPPH ethanol solution (0.03 mmol/L) and incubated in the dark for 30 min. Control groups included (1) deionized water + DPPH (control), (2) ethanol (blank), and (3) 0.1 mg/mL ascorbic acid (positive control). Absorbance at 517 nm was measured spectrophotometrically, with all experiments performed in triplicate. The DPPH free radical clearance was calculated using the following formula:

(4) $$DPPH\;scav(\%)=(1-({A_{s}-A}_{i})/A_{0})\times 100\%$$

where A_s_ is the absorbance of the sample at 517 nm; A_0_ is the absorbance of the control group; A_i_ is the absorbance of the blank group.

#### 2.7.2. ABTS Radical Scavenging Activity

The ABTS radical scavenging activity of curcumin, CLA-NLC, and Cur-CLA-NLC samples was evaluated using a modified ABTS assay [22]. ABTS solution (7 mmol/L) was mixed with potassium persulfate (2.45 mmol/L) in equal volumes and incubated in the dark for 12 h at room temperature. The mixture was then methanol-diluted to achieve an absorbance of 0.700 ± 0.02 at 734 nm. For testing, 4 mL of this solution was combined with a 0.15 mL sample, and the absorbance was measured at 734 nm after 10 min. Controls included deionized water (blank) and 1 mg/mL ascorbic acid (positive control). The ABTS radical scavenging rate was calculated by the following formula:

(5) $$ABTS\;scav(\%)\;=\;(1-{(A}_{s}/A_{0}))\;\times \;100\%$$

where A_s_ and A_0_ are the absorbance of the sample and the control at 734 nm, respectively.

#### 2.7.3. Ferric Reducing Antioxidant Power (FRAP)

The FRAP of curcumin, CLA-NLC, and Cur-CLA-NLC was determined using a modified method [23]. In brief, the mL sample was mixed with 2 mL phosphate buffer (pH 6.6, 0.2 mol/L) and 2 mL potassium ferricyanide (1%), then incubated at 50 °C for 20 min. After adding 2 mL trichloroacetic acid (10%), the mixture was centrifuged (5000 rpm, 10 min). Then, 2 mL supernatant was combined with 2 mL deionized water and 0.4 mL ferric chloride (0.1%), followed by dark incubation at 50 °C for 10 min. Finally, the absorbance of the sample at 700 nm was measured using a spectrophotometer.

### 2.8. Determination of Peroxide Value

Peroxide value (POV) serves as a key indicator of lipid oxidation, especially critical for encapsulated curcumin. Lipid-based delivery systems are susceptible to peroxidation, which can degrade curcumin and compromise its efficacy. Monitoring peroxide value is, therefore, essential to assess the long-term stability of Cur-CLA-NLC. Peroxide value was assayed according to the literature [24]. Briefly, 2 g of the sample was placed into a 250 mL conical flask, and 30 mL of the CHCl_3_-CH_3_COOH mixture solution was added and shaken to dissolve the sample. Then, add 0.5 mL of saturated KI solution, shake vigorously for 30 s, and place it away from light for 3 min. Next, titrating with 0.01 mol/L sodium thiosulfate until the solution in the bottle was light yellow. Finally, 0.5 mL starch indicator was added, and the titration continued with vigorous shaking until the blue color disappeared. Record the consumed volume of Na_2_S_2_O_3_ solution, and the peroxide value was calculated as follows:

(6) $$\mathrm{Peroxide}\;\mathrm{value}\;=(V-V_{0})\times c\times 0.1269/m\times 100$$

where V and V_0_ are the titrant volumes for the sample and blank, respectively; c is the titrant concentration; and m is the sample weight.

### 2.9. Cytotoxicity of Cur, CLA-NLC, and Cur-CLA-NLC

Cytotoxicity of Cur, CLA-NLC, and Cur-CLA-NLC on human normal liver cells L02 and human breast cancer cells MDA-MB-231 was determined by MTT assay (3-(4,5-dimethylthiazol-2-yl)-2,5-diphenyltetrazolium bromide assay). Cells cultured in Dulbecco’s Modified Eagle Medium with 10% fetal bovine serum and 1% penicillin–streptomycin (37 °C, 5% CO_2_) were seeded in 96-well plates (10^5^ cells/100 μL) and incubated for 24 h. After replacing the medium with 200 μL test samples (deionized water as blank) and incubating for 24 h, 20 μL MTT (5 mg/mL) was added per well. Following a 4 h incubation, the supernatant was removed, and formazan crystals were dissolved with 150 μL of dimethyl sulfoxide before measuring the absorbance at 490 nm. The cytotoxicity was evaluated according to the following formula [25]:

(7) $$\mathrm{Cell}\;\mathrm{viability}(\%)\;=\;A_{s}/A_{0}\;\times \;100\%$$

where A_s_ and A_0_ are the mean absorbance of the sample and the control group, respectively.

### 2.10. Statistical Analysis

This study utilized a completely randomized design, where samples were randomly assigned to experimental groups to ensure intergroup independence. Sample allocation was performed using SPSS 22.0′s random number generator, with each experimental group independently replicated three times (*n* = 3, unless stated specifically) while maintaining consistency of non-research factors. Data were expressed as the mean ± SD. Statistical analysis was performed using the one-way analysis of variance (ANOVA) with the software SPSS 22.0. The difference was recognized as significant at *p* < 0.05. All statistical assumptions (normality, homogeneity of variance, and independence) were verified.

## 3. Results and Discussion

### 3.1. The Construction of CLA-NLC and Cur-CLA-NLC

**Figure 1:**
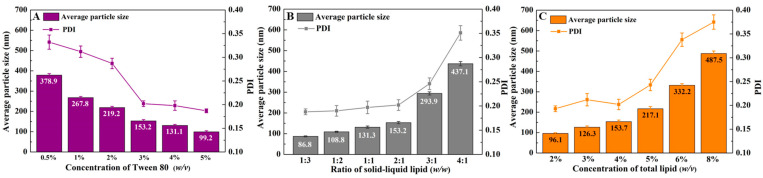
Particle size and PDI of CLA-NLC at different concentrations of Tween 80 (**A**), different mass ratios of solid–liquid lipid (**B**), and different total lipid concentrations (**C**) at 25 °C.

The classic preparation of NLC is essentially an emulsification of the lipid and drug in water with an emulsifier [26,27]. The emulsifier (Tween 80) dosage (Figure 1A), the ratio of stearic acid to CLA (Figure 1B), and total lipid concentration (Figure 1C) would influence the average particle and PDI of the CLA-NLC.

As Figure 1A shows, higher concentrations of Tween 80 (*w*/*v*) result in finer NLCs with narrower particle size distributions. When Tween 80 was added at 3% (*w*/*w*), the CLA-NLC particles exhibited a size of 153.2 nm with a PDI of 0.2. Considering cost and safety, 3% concentration of Tween 80 is selected for the subsequent experiment.

The correlation between the mass ratio of solid and liquid lipid (*w*/*w*) and CLA-NLC particle size is shown in Figure 1B. More liquid lipid resulted in a smaller particle size of CLA-NLC, which is attributed to the fact that liquid lipid reduces the surface tension and viscosity of the system, as found in similar studies [28,29]. However, over-loaded liquid lipid may also promote the drug release of the system. After comprehensive consideration, the mass ratio of solid and liquid lipid was selected as 2:1 to find a suitable total lipid concentration in the NLC (Figure 1C).

Figure 1C shows that the total lipid concentration in the NLC was positively correlated with the particle size of CLA-NLC, and the PDI value increased sharply when the lipid concentration was greater than 4% (*w*/*v*). This may be because when the total lipid concentration increased, the viscosity of the system became larger, resulting in easier aggregation between droplets, and the particle size of CLA-NLC also increased. The results of PDI and particle size were similar; that is, total lipid concentration was positively correlated with the PDI value. Numerous studies have also reported that increasing lipid content would result in higher PDI [30]. Therefore, considering process costs, stability of the CLA-NLC system, and curcumin encapsulation efficiency, the optimal total lipid concentration was determined to be 4%. Additionally, the CLA-NLCs in this study were prepared using both melt-emulsification and ultrasonic dispersion, a combination characterized by operational simplicity and low cost, demonstrating promising industrial applicability, particularly in functional foods.

### 3.2. Characterization and Thermal Stability of CLA-NLC and Cur-CLA-NLC

The FT-IR spectra of curcumin, CLA-NLC, and Cur-CLA-NLC are shown in Figure S1. It can be seen that 2915 cm^−1^ and 2848 cm^−1^ represent the antisymmetric and symmetric stretching vibration peaks of -CH_2_ in the CLA molecule, respectively. The absorption peak around 1450 cm^−1^ corresponds to the in-plane bending vibration absorption peak of -OH [14]. The peaks at 1428 cm^−1^ and 1377 cm^−1^ were enolic C=C-OH of curcumin. The FT-IR spectra of CLA-NLC and Cur-CLA-NLC are almost identical, and there is no characteristic absorption peak of curcumin in the spectra of Cur-CLA-NLC, which indicates that curcumin is well embedded in the lattice of CLA-NLC, so that its characteristic absorption peak is covered up. This is similar to the FT-IR results of lycopene nanostructured lipid carriers (Lyco-NLCs) composed of cholesterol and soybean lecithin reported in the literature [31].

With the UV-Vis calibration of curcumin concentration in ethanol solution (Figure S2A,B), the loading capacity of curcumin in the lipid phase is 862.4 μg/mL (93.6 μmol/L), calculated by the standard curve (Figure S2B). NLC has found its good position in enhancing the bioavailability and stability of hydrophobic bioactive compounds, such as curcumin. The NLC prepared with glycerol monostearate and medium-chain triglycerides has a high encapsulation efficiency of vitamin D3 [32]. In another case, a lipid phase formed by 0.1% of curcumin, 1.5% of phospholipon 90G (lecithin), 2.5% of medium chain triglycerides, and 2.5% of beeswax was emulsified in 1.5% of Tween 80 solution, and the suspension was finally dispersed in 10 volumes of cold water [26].

**Figure 2:**
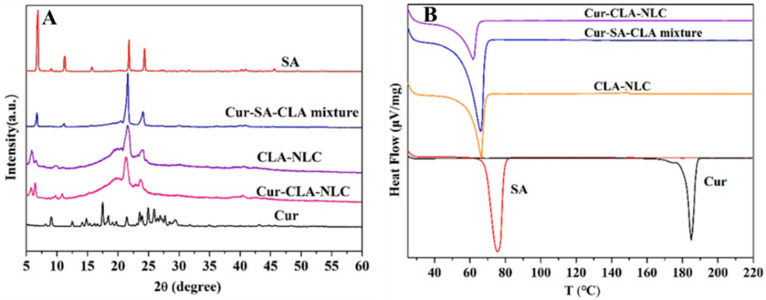
XRD patterns (**A**) and DSC curves (**B**) of SA, Cur, CLA-NLC, Cur-SA-CLA mixture, and Cur-CLA-NLC.

**Figure 3:**
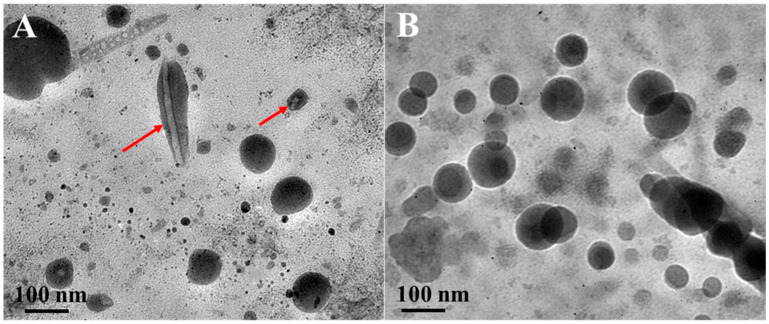
TEM images of CLA-NLC (**A**) and Cur-CLA-NLC (**B**). Conditions: 25 °C; Tween 80 concentration 3% (*w*/*v*); solid lipid: liquid lipid = 2:1 (*w*/*w*); total lipid concentration 4% (*w*/*v*).The red arrow indicates the fiber structure of lipids.

The XRD profile (Figure 2A) indicates that the CLA-NLC kept the crystal characteristics of SA, which was also confirmed by the TEM analysis of the CLA-NLC (Figure 3A). In addition, there is no diffraction peak of Cur in Cur-CLA-NLC, indicating that the diffraction peak of Cur is absent. The results show that curcumin may be dispersed in Cur-CLA-NLC nanostructured lipid carrier in an amorphous state or molecular state.

The thermal stability behavior of the lipid carrier was studied by DSC (Figure 2B), which would help to elucidate the mixing state of lipid and curcumin in the nanostructured lipid carrier. The initial transition temperature of solid lipid stearic acid (SA) was 68.99 °C, and its peak temperature was 75.71 °C. The melting peak corresponds to the melting of stearic acid, which was close to the melting point of stearic acid (67–72 °C). The initial transition temperature of the mixed lipids, the NLC, and the Cur-CLA-NLC was 60.37 °C, 66.04 °C, and 62.0 °C, respectively. The peak values were 66.51 °C, 56.61 °C, and 55.56 °C, indicating that curcumin encapsulation reduced the melting peak temperature of the Cur-CLA-NLC. Meanwhile, the endothermic melting peak of curcumin did not appear in the Cur-CLA-NLC system, which indicated that curcumin was well encapsulated in the NLC.

TEM images of nanostructured lipid carrier CLA-NLC and Cur-CLA-NLC are presented in Figure 3. It can be seen that the particle size of CLA-NLC is about 40–100 nm, and some of the nanoparticles show a fibrous structure inside (Figure 3A, red mark), referring to the stearic acid crystals. The Cur-CLA-NLC system (Figure 3B) is similar to CLA-NLC in terms of structure and particle size, indicating that curcumin-coated CLA-NLC still maintains a stable nanoparticle structure.

### 3.3. Bioaccessibility, Antioxidant Activity, and Anti-Lipid Peroxidation Ability of Cur-CLA-NLC

**Figure 4:**
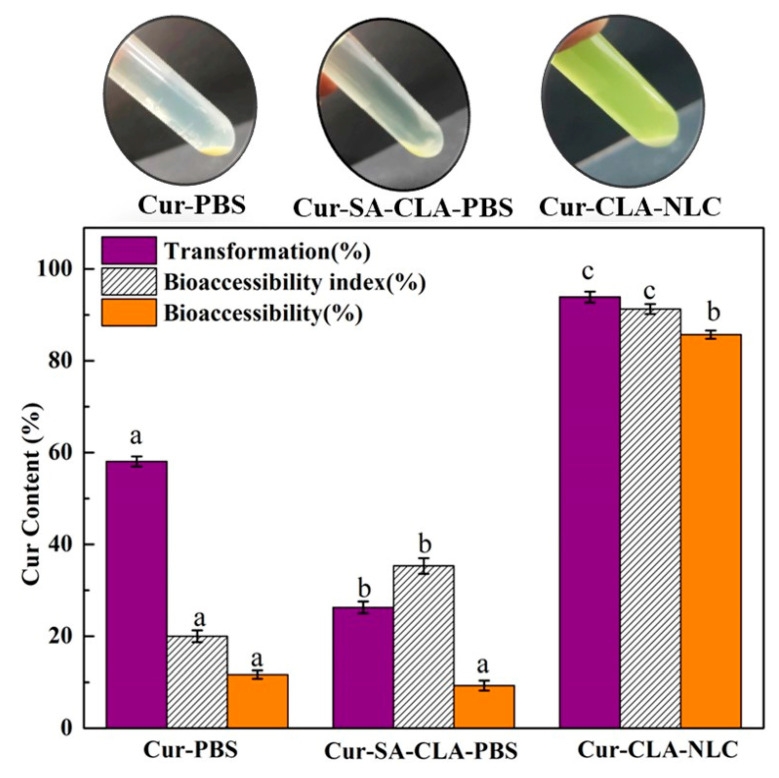
Bioaccessibility of Cur, the Cur-SA-CLA mixture, and Cur-CLA-NLC in simulated gastrointestinal digestive fluid. Conditions: 25 °C, 93.6 μmol/L of curcumin concentration. Different letters indicate statistically significant differences (*p* < 0.05).

Curcumin has a higher degradation rate and a lower bioaccessibility in the process of physiological metabolism [33]. Nanostructured lipid carrier is an effective delivery system that can be used to encapsulate bioactive substances and protect them from oxidation, heating, and other processes [34,35]. Hence, theoretically, the nanostructured lipid carrier system can be used to encapsulate the curcumin, which may improve its bioavailability while maintaining its original form. The bioaccessibility of curcumin, the Cur-SA-CLA mixture, and the Cur-CLA-NLC after digestion in simulated gastrointestinal fluid is shown in Figure 4.

After digestion of simulated gastroenteric fluid, the degradation rate of curcumin in CLA-NLC nanostructured lipid carriers was low, indicating good stability. The bioaccessibility of curcumin in the Cur-CLA-NLC system was 85.7%, which is much higher than that of the Cur-PBS system (11.7%) and the Cur-SA-CLA mixture system (9.3%). This was due to the insolubility of curcumin in water, the incomplete digestion of lipid in the Cur-SA-CLA mixture system, and the failure of the dissolved curcumin in lipid to release into the micellar phase, resulting in the low bioaccessibility of the system. However, in the Cur-CLA-NLC system, the smaller size of the nanostructured lipid carrier provides a larger surface area, enabling rapid lipase-mediated hydrolysis and release of free fatty acids. The free fatty acids generated during lipid digestion can promote the formation of mixed micelles and improve the dissolution of curcumin in intestinal fluid, thereby promoting digestion and enhancing the bioaccessibility of curcumin [36]. Meanwhile, the hydrophobic core of curcumin is stabilized by the fatty chain of CLA through van der Waals forces, a mechanism that can prevent premature degradation [37]. In summary, the bioaccessibility of Cur-CLA-NLC is 9.2 times higher than that of the Cur-SA-CLA mixture.

**Figure 5:**
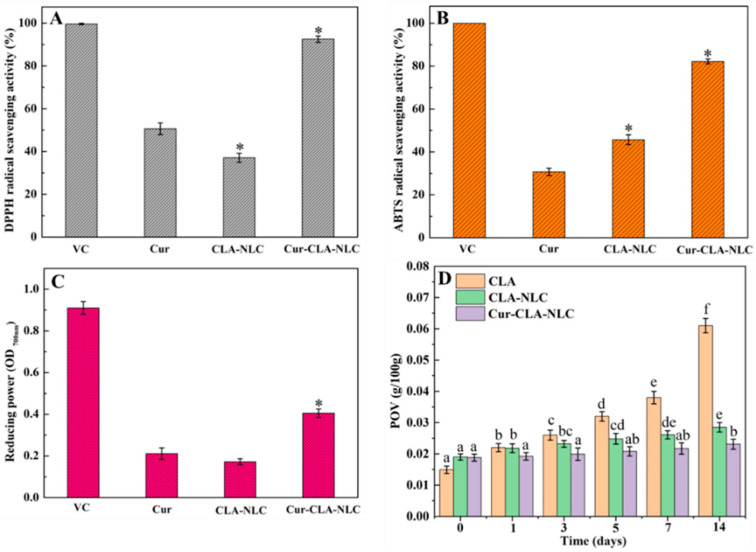
Antioxidant activity of Cur, CLA-NLC, Cur-CLA-NLC ((**A**): DPPH; (**B**): ABTS; (**C**): FRAP, * *p* < 0.05 compared with values of the Cur group) and (**D**) anti-lipid peroxidation ability of CLA, CLA-NLC, Cur-CLA-NLC. (37 °C, different letters represent different significant differences, *p* < 0.05).

The antioxidant activity results of Cur, CLA-NLC, and Cur-CLA-NLC are shown in Figure 5. It was surprising that Cur and CLA presented such a remarkable synergistic effect on all of the antioxidant activity measurements. Curcumin exhibited higher DPPH radical scavenging activity compared to both ABTS and FRAP assays. This is related to the structural advantages, differences in free radical properties, and sensitivity to reaction conditions of curcumin, which aligns with the study by Liu et al. [38]. At the same time, the ABTS radical scavenging activity of CLA-NLC (Figure 5B) was higher than Cur, while the DPPH scavenging capacity (Figure 5A) and FRAP (Figure 5C) were slightly lower than Cur, indicating that CLA had better ABTS scavenging activity. In the DPPH radical scavenging activity assay (Figure 5A), the radical scavenging rate of Cur-CLA-NLC was 92.5%, which was higher than that of Cur at 50.6% and CLA-NLC at 37.1%. The ABTS free radical scavenging rate (Figure 5B) of Cur-CLA-NLC is 82.3%, significantly higher than that of pure curcumin (30.7%) and CLA-NLC (45.6%). Similarly, the FRAP assay results demonstrate a comparable trend, with Cur-CLA-NLC exhibiting superior antioxidant activity. This is attributed to the formation of curcumin aggregates caused by low water solubility, which limits the interaction between curcumin and free radicals. The significant improvement in the free radical scavenging ability of curcumin after CLA-NLC loading is attributed to enhanced solubility mediated by the nanostructured lipid carrier. This is consistent with the research results reported in the literature [39].

In general, the antioxidant activity of Cur-CLA-NLC was significantly higher than that of CLA-NLC alone. These results indicated that CLA, as the liquid lipid of Cur-CLA-NLC, enhanced the antioxidant activity of the system. At the same time, CLA and Cur have significant synergistic antioxidant effects in the Cur-CLA-NLC system. The synergistic effect of CLA and curcumin may stem from their complementary antioxidant mechanisms: The molecular structure of curcumin contains phenolic hydroxyl groups that provide hydrogen atoms via hydrogen atom transfer, quenching free radicals by converting them into stable molecular compounds, and terminating the free radical chain reaction [40]. CLA, on the other hand, exhibits a unique ability to scavenge free radicals. The conjugated double bonds accept unpaired electrons from radicals, thereby neutralizing their reactivity [41]. Within the Cur-CLA-NLC, π-π stacking between curcumin’s phenolic rings and CLA’s conjugated dienes enhances electron delocalization efficiency, resulting in significantly higher antioxidant activity of the composite system compared to individual components. This is consistent with our previous research results in the Cur-OCLAVs-CS hydrogel system [14].

The anti-lipid peroxidation ability of CLA, CLA-NLC, and Cur-CLA-NLC is shown in Figure 5D. The peroxide values of all tested samples showed a gradual increase during storage, and the peroxide value of the CLA system increased most obviously. After 14 days, the peroxide value of CLA was about 4.1 times the initial value, while the peroxide value of CLA-NLC and Cur-CLA-NLC systems was only about 1.5 and 1.2 times the initial value, respectively. The results indicated that CLA-NLC and Cur-CLA-NLC had better anti-lipid peroxidation ability than CLA alone. This is attributed to the encapsulation of liquid lipid within solid lipid in the NLC system, thereby reducing the oxidation of CLA in CLA-NLC. In addition, the presence of curcumin further improves the anti-lipid oxidation capacity of the Cur-CLA-NLC system, and the peroxide value of CLA and CLA-NLC after 14 days was 2.7 and 1.2 times that of Cur-CLA-NLC, respectively. This is attributed to the fact that curcumin not only has the ability to resist lipid oxidation, but also the combined effect with CLA further enhances the anti-lipid oxidation capacity of the system.

### 3.4. The Cytocompatibility of CLA-NLC and Cur-CLA-NLC

**Figure 6:**
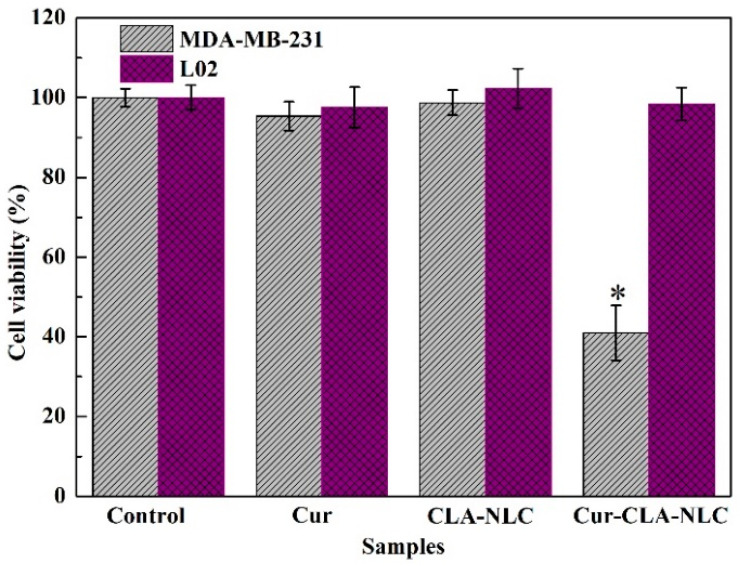
Cell viability of Cur, CLA-NLC, and Cur-CLA-NLC on L02 and MDA-MB-231. Conditions: 25 °C, 93.6 μmol/L of Cur concentration. Values are presented as means ± standard deviation, *n* = 3. * *p* < 0.05 compared with values of the control group.

Subsequently, we studied the biocompatibility of the Cur-CLA-NLC system and its inhibitory effect on breast cancer cells MDA-MB-231. The cell viability of curcumin, CLA-NLC, and Cur-CLA-NLC on human normal liver cells (L02) and human breast cancer cells (MDA-MB-231) is shown in Figure 6. All the samples showed no cytotoxicity to human normal liver cell L02, indicating their good biocompatibility. At the same time, CLA-NLC had no cytotoxicity on MDA-MB-231, while Cur-CLA-NLC showed an obvious inhibitory effect on MDA-MB-231 with a cell viability of 40.98% after 24 h of culture.

The results showed that the prepared Cur-CLA-NLC had good biocompatibility and could also significantly improve the inhibitory effect of curcumin on human breast cancer cells MDA-MB-231. This is because curcumin, as a natural polyphenol compound, inhibits proliferation and migration while inducing apoptosis in breast cancer cell MDA-MB-231 through a multi-target mechanism [42]; Meanwhile, CLA modulates tumor occurrence, promotion, and metastasis via lipid peroxidation, fatty acid composition regulation, and cell cycle disruption [43,44]. Therefore, in Cur-CLA NLCs, the combined action of curcumin and CLA suppresses angiogenesis and promotes apoptosis in MDA-MB-231 cells by targeting multiple signaling pathways. This is similar to the results reported in the literature study: that is, compared with pure curcumin, the Cur-NLC demonstrates excellent anti-cancer activity by inhibiting proliferation and inducing apoptosis in human HepG2 cells [45]. Therefore, this indicates that the prepared Cur-CLA-NLC system has good biocompatibility and can significantly enhance the inhibitory effect of Curcumin on human breast cancer MDA-MB-231 cells.

## 4. Conclusions

In this study, curcumin was encapsulated in nanostructured lipid carriers (Cur-CLA-NLC) constructed using conjugated linoleic acid (CLA) as the liquid lipid and stearic acid (SA) as the solid lipid. The particle size of CLA-NLC decreased with the increase in the concentration of Tween 80 and the proportion of liquid lipid. Finally, the optimal conditions of 3% (*w*/*v*) concentration of Tween 80, 2:1 ratio of solid to liquid lipid, and 4% (*w*/*v*) concentration of total lipid were determined. FT-IR, XRD, and DSC results show that curcumin is well encapsulated in nanostructured lipid carrier CLA-NLC. At the same time, curcumin did not affect the thermal stability of Cur-CLA-NLC, and curcumin was dispersed in CLA-NLC nanostructured lipid carrier in an amorphous state or molecular state. In vitro simulated digestion studies have shown that the Cur-CLA-NLC system significantly increases the bioavailability of curcumin. The bioavailability of curcumin in the Cur-CLA-NLC system is 85.7%, which is 7.3 times higher than that of pure curcumin and 9.2 times higher than that of the Cur-SA-CLA mixture.

The antioxidant activity results showed that CLA, as the liquid lipid in Cur-CLA-NLC, enhanced the antioxidant activity of the system and exhibited significant synergistic antioxidant effects with curcumin. In addition, the Cur-CLA-NLC system can reduce the peroxide value, improve the anti-lipid oxidation ability of CLA, and show good biocompatibility. Therefore, Cur-CLA-NLC serves as a promising carrier for curcumin delivery. However, the constructed Cur-CLA-NLC system exhibits relatively low drug-loading capacity and potential long-term stability issues, such as lipid crystallization or curcumin leakage. Future research could leverage AI-guided predictive models to identify optimal lipid compositions, thereby enhancing both loading efficiency and stability of curcumin. The Cur-CLA-NLC delivery system can be incorporated into functional foods as an additive for functional beverages or an enhancer for dairy products.

## Data Availability

Data is contained within the article or Supplementary Material. Further inquiries can be directed to the corresponding authors.

## Supplementary Materials

The following supporting information can be downloaded at: https://www.mdpi.com/article/10.3390/foods14173104/s1. Figure S1: FT-IR spectra of Cur, CLA-NLC, and Cur-CLA-NLC; Figure S2: UV-Vis absorption spectrum of curcumin in ethanol solution (A) and standard curve of curcumin at 427 nm in SA-CLA mixed lipid phase (B).

## Abbreviations

The following abbreviations are used in this manuscript:

ABTS	2,2′-azino-bis (3-ethylbenzothiazoline-6-sulfonic)
CLA	Conjugated linoleic acid
Cur	Curcumin
DPPH	1,1-Diphenyl-2-picryl hydrazyl radical
DSC	Differential Scanning Calorimeter
FRAP	Ferric reducing antioxidant power
FT-IR	Fourier infrared spectroscopy
MTT	3-(4,5-dimethylthiazol-2-yl)-2,5-diphenyltetrazolium bromide
NLC	Nanostructured lipid carrier
PBS	Phosphate-Buffered Saline
PDI	Polydispersity index
SA	Stearic acid
TEM	Transmission Electron Microscope
UV-Vis	Ultraviolet–visible spectroscopy
XRD	X-ray diffraction

## References

[1] Sun Q., Lv M., Li Y. (2024). Nanotechnology-based drug delivery systems for curcumin and its derivatives in the treatment of cardiovascular diseases. J. Funct. Foods.

[2] Ma P., Zhang Z., Tsai S., Zhang H., Li Y., Yuan F., Wang Q. (2021). Curcumin-loaded pickering emulsion formed by ultrasound and stabilized by metal organic framework optimization. Foods.

[3] Liu Q., Chen T., Chen L., Zhao R., Ye X., Wang X., Wu D., Hu J. (2024). High Internal Phase Emulsions Stabilized with Ultrasound-Modified Spirulina Protein for Curcumin Delivery. Foods.

[4] Karaca A.C., Rezaei A., Qamar M., Assadpour E., Esatbeyoglu T., Jafari S.M. (2025). Lipid-based nanodelivery systems of curcumin: Recent advances, approaches, and applications. Food Chem..

[5] Ghasemi M.A.G., Hamishehkar H., Javadi A., Homayouni-Rad A., Jafarizadeh-Malmiri H. (2024). Natural-based edible nanocomposite coating for beef meat packaging. Food Chem..

[6] Kaewmalun S., Yata T., Kitiyodom S., Yostawonkul J., Namdee K., Kamble M.T., Pirarat N. (2022). Clove oil-nanostructured lipid carriers: A platform of herbal anesthetics in Whiteleg Shrimp (*Penaeus vannamei*). Foods.

[7] Feng J., Wang Z., Huang W., Zhao X., Xu L., Teng C., Li Y. (2025). Hyaluronic acid-decorated lipid nanocarriers as novel vehicles for curcumin: Improved stability, cellular absorption, and anti-inflammatory effects. Food Chem..

[8] Xu Y., Li X., Dai Z., Zhang Z., Feng L., Nie M., Liu C., Li D., Zhang M. (2023). Study on the relationship between lutein bioaccessibility and in vitro lipid digestion of nanostructured lipid carriers with different interface structures. Food Hydrocoll..

[9] Wang X., Sun J., Zhao S., Zhang F., Meng X., Liu B. (2023). Highly stable nanostructured lipid carriers containing can-delilla wax for d-limonene encapsulation: Preparation, characterization and antifungal activity. Food Hydrocoll..

[10] Viegas C., Patrício A.B., Prata J.M., Nadhman A., Chintamaneni P.K., Fonte P. (2023). Solid lipid nanoparticles vs. nanostructured lipid carriers: A comparative review. Pharmaceutics.

[11] Zaky M.F., Megahed M.A., Hammady T.M., Gad S., Ghorab M.M., El-Say K.M. (2022). Tailoring apixaban in nanostructured lipid carrier enhancing its oral bioavailability and anticoagulant activity. Pharmaceutics.

[12] Cakir N., Altas B.O., Kalaycioglu G.D., Mustafaoglu N., Aydogan N. (2025). Development and Characterization of DHA-Integrated Nanostructured Lipid Carrier Formulations for Enhanced Cellular Binding and Uptake. Colloids Surf. Physicochem. Eng. Aspects.

[13] La Torre C., Caputo P., Cione E., Fazio A. (2024). Comparing nutritional values and Bioactivity of Kefir from different types of animal milk. Molecules.

[14] Liu H., Meng X., Li L., Xia Y., Hu X., Fang Y. (2023). The incorporated hydrogel of chitosan-oligoconjugated linoleic acid vesicles and the protective sustained release for curcumin in the gel. Int. J. Biol. Macromol..

[15] Park S.J., Garcia C.V., Shin G.H., Kim J.T. (2018). Improvement of curcuminoid bioaccessibility from turmeric by a nanostructured lipid carrier system. Food Chem..

[16] Moraes S., Marinho A., Lima S., Granja A., Araújo J.P., Reis S., Sousa C.T., Nunes C. (2021). Targeted nanostructured lipid carriers for doxorubicin oral delivery. Int. J. Pharm..

[17] Behbahani E.S., Ghaedi M., Abbaspour M., Rostamizadeh K., Dashtian K. (2019). Curcumin loaded nanostructured lipid carriers: In vitro digestion and release studies. Polyhedron.

[18] Liu H., Meng X., Li L., Hu X., Fang Y., Xia Y. (2021). Synergistic effect on antioxidant activity of vitamin C provided with acidic vesiculation of hybrid fatty acids. J. Funct. Foods.

[19] Madhavi B.G.K., Wijethunga A.M., Okagu O.D., Sun X. (2024). Defatted Wheat Germ Protein-Derived Peptides Showed Multiple Biological Activities from the Stomach to Small Intestine: In Silico and In Vitro Approaches. J. Agr. Food Chem..

[20] Carpenter J., George S., Saharan V.K. (2019). Curcumin encapsulation in multilayer oil-in-water emulsion: Synthesis using ultrasonication and studies on stability and antioxidant and release activities. Langmuir.

[21] Mengíbar M., Miralles B., Heras Á. (2017). Use of soluble chitosans in Maillard reaction products with β-lactoglobulin. Emulsifying and antioxidant properties. LWT.

[22] Fan Y., Liu Y., Gao L., Zhang Y., Yi J. (2018). Oxidative stability and in vitro digestion of menhaden oil emulsions with whey protein: Effects of EGCG conjugation and interfacial cross-linking. Food Chem..

[23] Kchaou H., Benbettaïeb N., Jridi M., Abdelhedi O., Karbowiak T., Brachais C.-H., Léonard M.-L., Debeaufort F., Nasri M. (2018). Enhancement of structural, functional and antioxidant properties of fish gelatin films using Maillard reactions. Food Hydrocoll..

[24] Luo C., Wu B., Lin Z., Yuan L., Wang H. (2013). Determination of Peroxide Value in the Plastic Resin. Asian J. Chem..

[25] Sohail R., Abbas S.R. (2020). Evaluation of amygdalin-loaded alginate-chitosan nanoparticles as biocompatible drug delivery carriers for anticancerous efficacy. Int. J. Biol. Macromol..

[26] Gonçalves R.F., Vicente A.A., Pinheiro A.C. (2023). Incorporation of curcumin-loaded lipid-based nano delivery systems into food: Release behavior in food simulants and a case study of application in a beverage. Food Chem..

[27] Wu X., Zhang J., Wang M., Sun Z., Chang C., Ying Y., Li D., Zheng H. (2025). Effect of emulsifier type on camellia oil-based nanostructured lipid carriers for delivery of curcumin. Food Chem..

[28] Jaiswal P., Gidwani B., Vyas A. (2016). Nanostructured lipid carriers and their current application in targeted drug delivery. Artif. Cells Nanomed. Biotechnol..

[29] Chideme N., De Vaal P.L. (2024). Effect of liquid viscosity and surface tension on the spray droplet size and the measurement thereof. J. Appl. Fluid Mechan..

[30] Velmurugan R., Selvamuthukumar S. (2016). Development and optimization of ifosfamide nanostructured lipid carriers for oral delivery using response surface methodology. Appl. Nanosci..

[31] Ma Y., Li C., Xiu W., Wang X. (2023). In vivo and in vitro evaluation of stability and antioxidant activity of lyco-pene-nanostructured lipid carriers. Food Sci. Biotechnol..

[32] Azevedo M.A., Cerqueira M.A., Gonçalves C., Amado I.R., Teixeira J.A., Pastrana L. (2023). Encapsulation of vitamin D3 using rhamnolipids-based nanostructured lipid carriers. Food Chem..

[33] Li R., Lin Z., Zhang Q., Zhang Y., Liu Y., Lyu Y., Li X., Zhou C., Wu G., Ao N. (2020). Injectable and in situ-formable thiolated chitosan-coated liposomal hydrogels as curcumin carriers for prevention of in vivo breast cancer recurrence. ACS Appl. Mater. Interf..

[34] Kovačević A.B., Müller R.H., Keck C.M. (2020). Formulation development of lipid nanoparticles: Improved lipid screening and development of tacrolimus loaded nanostructured lipid carriers (NLC). Int. J. Pharm..

[35] Hashemi F.S., Farzadnia F., Aghajani A., Ahmadzadeh NobariAzar F., Pezeshki A. (2020). Conjugated linoleic acid loaded nanostructured lipid carrier as a potential antioxidant nanocarrier for food applications. Food Sci. Nutr..

[36] Ortega N., Reguant J., Romero M.-P., Macià A., Motilva M.-J. (2009). Effect of fat content on the digestibility and bioac-cessibility of cocoa polyphenol by an in vitro digestion model. J. Agr. Food Chem..

[37] Tian Y., Wang N., Liu H., Qiu T., Chen C., Liu X., Zhu Y. (2025). Extraction of curcuminoids from *Curcuma longa* L. by fatty acid-based ionic liquid aqueous solution: Experimental and mechanism study. Food Chem..

[38] Liu Q., Xie M., Li X., Song Y., Wang Y., Hong P., Zhou C. (2024). Development of curcumin-loaded hyaluronic acid complexes for curcumin intestinal delivery: Preparation, characterization, antioxidant, and in vitro digestive properties. LWT.

[39] Cheng Q., Liu C., Zhao J., Guo F., Qin J., Wang Y. (2024). Hyaluronic acid modulates techno-functional and digestion properties of heat-induced ginkgo seed protein isolate gel. Food Biosci..

[40] Wu H., Liu Z., Zhang Y., Gao B., Li Y., He X., Sun J., Choe U., Chen P., Blaustein R.A. (2024). Chemical composition of turmeric (*Curcuma longa* L.) ethanol extract and its antimicrobial activities and free radical scavenging capacities. Foods.

[41] Wu Z., Chen H., Yang B., Zhao J., Chen W. (2024). Structural identification and antioxidant activity of trans-9, trans-11, cis-15-conjugated linolenic acid converted by probiotics. Food Res. Int..

[42] Ros M., Riesco-Llach G., Polonio-Alcalá E., Morla-Barcelo P.M., Ruiz-Martínez S., Feliu L., Planas M., Puig T. (2024). Inhibition of Cancer stem-like cells by Curcumin and other polyphenol derivatives in MDA-MB-231 TNBC cells. Int. J. Mol. Sci..

[43] Du M., Gong M., Wu G., Jin J., Wang X., Jin Q. (2024). Conjugated linolenic acid (CLnA) vs conjugated linoleic acid (CLA): A comprehensive review of potential advantages in molecular characteristics, health benefits, and production techniques. J. Agric. Food Chem..

[44] Beatty A., Singh T., Tyurina Y.Y., Tyurin V.A., Samovich S., Nicolas E., Maslar K., Zhou Y., Cai K.Q., Tan Y. (2021). Ferroptotic cell death triggered by conjugated linolenic acids is mediated by ACSL1. Nat. Commun..

[45] Wang F., Ye X., Zhai D., Dai W., Wu Y., Chen J., Chen W. (2020). Curcumin-loaded nanostructured lipid carrier induced apoptosis in human HepG2 cells through activation of DR5/caspases-mediated extrinsic apoptosis pathway. Acta Pharm..

