# Supplementary material for: Development of Conjugated Linoleic Acid Nanostructured Lipid Carriers and Their Synergistic Efficacy with Curcumin
Source: Foods. 2025 Sep 5;14(17):3104. doi: 10.3390/foods14173104 (PMC12427970; doi:10.3390/foods14173104)
Supplement: Supplementary file 1 [file foods-14-03104-s001.zip › foods-3816751-supplementary.pdf]

A healthy fatty acids-nanostructured lipid carrier and its synergistic effect with encapsulated curcumin

Supporting information

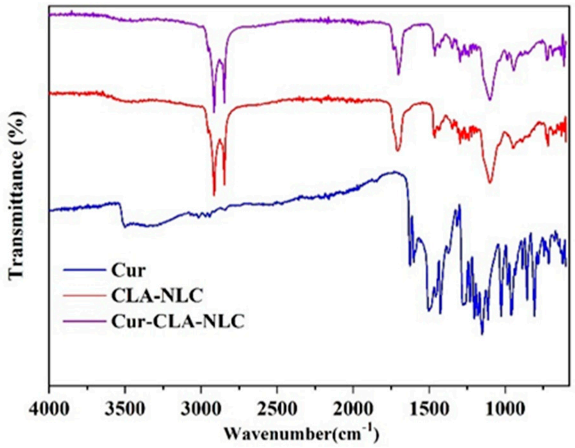

Figure S1. FT-IR spectra of Cur, CLA-NLC and Cur-CLA-NLC.

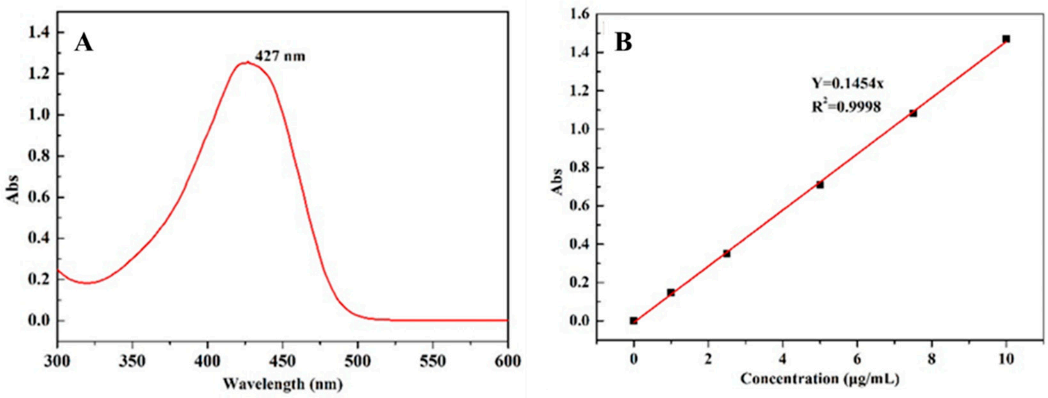

Figure S2. UV absorption spectrum of curcumin in ethanol solution (A) and standard curve of curcumin at 427nm in SA-CLA mixed lipid phase (B).
